# Incorporating Participatory Action Research in Attention Bias Modification Interventions for Addictive Disorders: Perspectives

**DOI:** 10.3390/ijerph16050822

**Published:** 2019-03-06

**Authors:** Melvyn W.B. Zhang, Jiangbo Ying

**Affiliations:** 1 National Addiction Management Service, Institute of Mental Health, Singapore 539747, Singapore; yingjiangbo@gmail.com; 2 Lee Kong Chian School of Medicine, Nanyang Technological University, Singapore 380322, Singapore

**Keywords:** participatory design research, co-design, qualitative, attention bias, cognitive bias, addiction

## Abstract

Participatory action research was introduced in the 1960s and early 1970s, but it has only been more widely adopted in the recent years. Such methodologies have since been applied to several web & mobile-based interventions in psychiatry. To date no prior review has scoped the extent of the application of such methodologies for web & mobile-based interventions in psychiatry. In this article, a scoping literature review was performed, and seven articles have been identified. The most common methodologies are that of co-design workshops; and increasingly service users and participants are included in these workshops. There remains a lack of application of such methodologies for addiction research. Increasingly, attention and cognitive bias modification interventions are more commonplace, given that they have been found to be effective in modifying underlying biases amongst individuals with addictive disorders. Unfortunately, there remains to be inherent limitations with web and mobile versions of attention and cognitive bias modification interventions. Participatory design research methods could help address these limitations and future research involving the conceptualization of new attention or cognitive bias modification applications ought to consider the incorporation of these research methods.

## 1. Overview of Participatory Design Research

Participatory action research was introduced in the 1960s and early 1970s. However, it was only in the last decade that there has been more focus on the utilisation of these research methodologies [1,2,3]. A recent review [4], conducted in 2008, looked at the uptake and design of participatory design research conducted in nursing research between the years of 2000 through to 2005 and highlighted that at least 38% of the identified published research studies have included such methodologies. Of these studies, which included participatory research methodologies, 13% were clinical studies. Thus, there is clear evidence that such research methodologies have been more widely used in the last decade. Munn-Giddings et al. 2008 [4] review also reported there being a diversity in the research methods utilised. More importantly, their review also highlighted that most of these prior studies included only practitioners, instead of other healthcare professionals or patients. This is particularly concerning, as it implies that the opinions and perspectives of other key stakeholders might not be captured. 

According to Green et al. [5], participatory action research refers to a process of “systematic inquiry, with the participation of those affected by the problem being studied, for the purposes of education and action or effecting social change.” Participatory design research typically is qualitative in nature, and it involves key stakeholders in the research process, as it is believed that the inclusion of key stakeholders (such as patients), would increase the relevance of the research, and increase the acceptability of the findings generated [6]. The application of participatory research methods thus involves a collaboration with relevant stakeholders in exploring the following aspects of the research: developing and defining core research questions; the conduct of the research, collation of data, dissemination of data and findings [6]. Such methods have been increasingly applied for psychiatry and mental health research. In psychiatry, the conventional methodologies utilized are that of community-based participatory research, participatory action research, participatory design and lastly, user-centered design [6]. Participatory action research and participatory community research are similar, as both methods involve key participants or community members, to result in change either at the individual, group or community level [7]. Participatory design research involves iterative design cycles with participants [7]. The user-centred design approach is usually driven by either researchers or designers. In this approach, the participants’ inputs are sought for, which will help shape the researchers’ plan for the eventual research design [7]. In the recent years, technological solutions, such as E-Health and M-Health have been widely used in psychiatry, as they helped to reduce geographical barriers, increased accessibility and potentially help in the reduction of the cost of various interventions [8]. E-Health refers to electronic health and includes web-based and internet-based interventions [8]. M-Health refers to mobile health and includes smartphone-based interventions, mainly in the form of smartphone applications [9]. 

Participatory methodologies have been increasingly used to aid the development and refinement of various web-based and mobile-based interventions. O’Conner et al. [10] recognised that many of the existing smartphone applications had not met the specific needs of individuals living with dementia, nor the needs of their caregivers. Hence, they utilised a focus group approach to identify barriers experienced by participants, in order to guide the co-design process. Ospina-Pinillos et al. [11] in their study utilised participatory design research methodologies, involving young individuals aged between 16 and 25 years old and youth health professionals, to facilitate the development of a web-based mental health clinic. Such a web-based mental health clinic will help to provide young people with timely access to mental health care. 

## 2. Application of Participatory Design in Research in Other Disciplines

Particiapatory research methodologies have been commonly applied in various areas of research in other disciplines. Haijes et al. [12] reviewed the application of participatory methods for pediatric research. In their review, a total of 24 articles were identified, and amongst these studies, methods applied included that of descriptive, verbal, written and active methods. Hajies et al. [12] in their review highlighted that the application of participatory methods were limited primarily to data collation, and there remains a gap in the use of such methodologies for collaborative participation between the youths and researchers. Brett et al. [13] in their review sought to determine the implications that participatory research methods have had on different individuals, ranging from service users, to researchers and communities. Whilst Brett et al.’s [13] review was not focused primarily on any specific domains in medicine, they reported that the inclusion of participatory design methods help increased users’ empowerment. Nevertheless, the inclusion of participatory methods were hindered at times, due to service users not being adequately trained, or due to the lack of funding for this nature of research [13]. Apart from the application of such methods in pediatric research, these methods are also appropriate when considering a geriatric population. Blair et al. [14], in their prior review identified 10 studies, in which these methods have been applied. Participatory research methods have been used in aiding researchers in understanding vitality in older individuals; perceptions of elder abuse; in enhancing stroke services; exploring health and social needs; exploring perceptions of fall risk and for determining the means of health assessment [14]. It is clear from these reviews that participatory design research has been considered for different subpopulations and it has since been utilised across a variety of research, exploring different needs or medical conditions. 

## 3. Application of Participatory Design Research for Web & Mobile-Based Interventions in Psychiatry

As mentioned in the Introduction, participatory design research has also been increasingly used in medicine. The application of these methods are also not confined to medical disciplines, but there have been studies reporting the use of these methods in psychiatry. However, there remains to date, a lack of understanding as to how such methods have been applied for web-based and mobile-based interventions in psychiatry. Given this, in the first part of this short communication article, we will briefly provide an overview of the literature of how these methods have been applied in psychiatry. In the second part of this article, we will provide an overview of attention biases and its theoretical underpinnings, and an overview of attention bias modification interventions and their inherent limitations. We will conclude with our perspectives on how participatory design research methodologies could be applied for attention bias modification interventions; and how such methods might address some of the existing limitations of current attention bias modification interventions. 

A search was conducted through to 19 September 2018, on the following databases: PubMed, MEDLINE and PsycINFO. The following search terminologies were used in the search process: (“participatory design” OR “co-design” OR “co-operative design” OR “co-creat*”) AND (“psychiatry”) AND (“e-health” OR “ehealth” OR “m-health” OR “mhealth” OR “mobile health” OR “telehealth” OR “telemedicine” OR “telemedicine”). Articles were extracted if they have utilised participatory research design and co-design in their methods and if they focused on the creation of an E-health or M-health intervention, which was specific for mental health. Articles were excluded if they did not mention that they have adopted any form of participatory research design or co-design in their methods; and if the focus was on the creation of an E-health or M-health intervention for other medical conditions. A total of 389 citations were identified from PubMed, MEDLINE and PsycINFO. Two hundred and thirty-three duplicated articles were removed, leaving 156 citations. On further screening, 112 citations were excluded as they were not relevant. Forty-four full-text articles were downloaded. Of these forty-four full-text articles, thirty-seven full-text articles were excluded, of which one article was a protocol paper and thirty-six were not focused on mental health conditions. Our search found seven articles that described the application of participatory design research for such interventions. Table 1 provides an overview of the characteristics of the identified studies. 

Only five articles out of the seven identified articles explicitly mentioned the psychiatric condition that they targeted, which included that of perinatal depression, dementia, self-harm, general mental health well-being of males and youth mental health issues. Ospina-Pinillos et al. [11] and Wiljer et al. [19] did not specify the psychiatric condition they were targeting and in both these studies, the objective of including participatory action research methodologies was to facilitate the co-creation of a mental health intervention. Whilst there were variations in the methods of participatory action research used across the studies, the most common methods were that of design or co-creation workshops and focus groups. Not all the studies that we have identified have detailed the methodology of analysis of the data obtained from the design or co-creation workshop and focus groups. Different studies provided different rationales for the inclusion of participatory design research methods. O’Connor et al. [10] considered the utilisation of a qualitative exploratory case study design involving focus groups, in order to elucidate all the potential barriers that participants might face. This helps to ensure that the eventual application that is developed is more effective. Owens et al. [16] highlighted the importance of including participatory design research methods in their study, as it helps to ensure that the text messages (which are delivered to individuals with self-harm tendencies) were safe and personalised. Both Whitehouse et al. [18] and Wilier et al. [19] have highlighted that the adoption of participatory design approaches would help to improve uptake of interventions that are created. It is thus evident from the literature review that participatory design research has not only been increasingly adopted and has been harnessed upon to help inform the conceptualization process and to also increase uptake, acceptability and usability of the eventual developed research product. These articles highlight the importance of considering such research methodologies in the conceptualization of new web-based or mobile-based interventions. It is also evident that such methods have been applied only for a selected few psychiatric disorders. 

The identified studies also revealed there is diversity regarding the participants who were invited to the co-creation or design ideation workshops, whilst in Munn-Giddings et al.’s [4] review, they have highlighted that only practitioners were typically invited, our current literature review highlights that there have been more studies which have considered the inclusion of service users and participants. It is of importance to consider the needs of participants and service users, given that they are eventually going to be ones who are using the intervention. 

## 4. Attention Bias Modification & Its Clinical Significance for Addictive Disorders

Whilst conventional psychotherapies like cognitive behavioral therapy have mainly been focused on modifying reflective, conscious processes, attention bias modification involves the modification of unconscious, automatic processes, that of attentional biases. Attentional biases refer to the preferential allocation of attentional processes towards substance-related cues or stimuli in the natural environment [20]. Closely related to attentional biases are approach biases, which refer to automated predisposed tendencies for individuals to reach out and approach substance-related stimuli [21]. The theoretical underpinning attentional and approach biases is that of the dual-process theory, that posits that with the repeated exposure to a substance, this results in a corresponding increment in the automatic processing of such cues and an inhibition of the normal cognitive control processes [22]. Attentional bias modification is typically achieved by means of reaction time tasks, that of the visual probe task or the Stroop task. When assessing for the presence or absence of attentional biases, for the visual probe task, this involves probes that replaces the neutral (non-substance related image) and the substance image at equal frequencies. However, when the visual probe task is used in the modification of attentional biases, this involve probes always replacing the neutral image, in order to retrain attentional processes away from the substance-related stimuli [22]. With the advances in the field of experimental psychology, there has since been extensive evaluation of attention bias modification. Cristea et al. [22] in their review, which included studies involving participants with alcohol and tobacco use disorder reported bias modification to be moderately effective in reduction biases, with an overall effect size that of 0.60. Other studies [23] have critiqued Cristea et al.’s [22] findings, as their review synthesized a mixture of clinical and laboratory studies. Thus, there remains doubts pertaining to the effect size, given that heterogenous studies were considered for the synthesis. In a commentary published recently [23] in response to Cristea et al.’s [22] study, bias modification remains to be effective when only clinical studies are considered in the synthesis. In another study, individuals with opioid dependence and who were maintained on methadone, undertook an attention bias modification intervention, and there was not only a reduction in attentional biases, but also that of cravings, dosing of medications administered and relapses [24]. Similarly, Manning et al. [25] reported of the effectiveness of cognitive bias modification amongst their group of inpatients who undertook the program, whilst undergoing medication-assisted detoxification. These studies thus provide evidence for the effectiveness of bias modification amongst individuals with addictive disorders. In addition, these studies also provide evidence for the presence of such unconscious processes in individuals with addictive disorders, that could predispose them to a slip or relapse back into their addictive behaviors. 

## 5. Web & Mobile-Based Attention Bias Modification Interventions

Jones et al. [26] in their review of meta-analyses reported that bias modification was particularly effective when delivered within the confines of the laboratory. This is not unexpected, given that a laboratory setting would allow for greater supervision and compliance to the task [26] However, the advances in both web and mobile-based technologies has led to there being more of such interventions being delivered remotely, out of the confines of the laboratory. Wittekind et al. [27] reported how they had made use of web-technologies to deliver approach and avoidance bias modification intervention for smoking, and this has led to a reduction in cigarette consumption, dependence and drive amongst their participants. In Wittekind CE et al.’s [27] prior study, the conceptualization of the bias modification intervention was based on the principles of the conventional approach and avoidance training task. The development of the intervention was by means of a computer program, that of Visual Basic2010 Express. There was no mention of how academics have collaborated with participants in the development of such an intervention. Zhang et al. [28] in their systematic review and content analysis of attention and cognitive bias modification applications from the published literature and the commercial stores, reported that being demonstrated effectiveness of mobile-based bias modification in seven out of the eight published studies. Of which, one of the applications (Chimpshop) which is also commercially available, has been evaluated previously and was effective in reducing drinking in individuals by up to 60% [29]. Yang et al. [30] highlighted the inherent advantages of harnessing mobile health technologies for bias modification interventions. Delivering attention bias modification intervention by means of a mobile application allows for training to be conducted in diverse locations [30]. This allows for the generalization of clinical benefits and increases the frequency of training [30]. The increased frequency of training could potentially lead to better and improved clinical outcomes [30]. Moreover, there are advantages of a mobile variant of such tasks over web-based variant, as the mobile variant does not require individuals to be consistently connected to the Internet to undertake the bias modification task. 

While the prior review by Zhang et al. [28] has highlighted the effectiveness of mobile-based bias modification; there remain to challenge intrinsic to such psychological (attention bias modification) task. Attention bias is typically being modified using either the Stroop task or the visual probe task, and participants are typically required to complete numerous repeats of the trials, to effect a change in their underlying biases. Thus, one of the challenges intrinsic to attention bias modification is that of the participants’ motivation to train, given that the bias modification tasks tend to be highly repetitive, and with a need for multiple training sessions. Gamification, which refers to the use of game-design features in a non-gaming context has been considered to address the issues of declining motivation to train. Zhang et al. [31] in their review of gamified attention bias interventions highlighted that only 50% of the identified studies reported a gamified application to be effective, and the studies which reported effectiveness were conducted by the same research group, using the same application. In Zhang et al.’s [31] review, they have highlighted Boendermaker et al. [32]’s study, which used a shot game for attention bias modification amongst individuals with alcohol use problems. Of significance, the gamified task did not reduce attention bias and even failed to achieve a decline in the overall levels of alcohol consumption [32]. Thus, it is especially pertinent to examine the reasons as to why gamification works in some studies, but not others. 

## 6. Application of Participatory Design Research Methods for Mobile Attention Bias Modification Interventions

Our brief review has highlighted the increased adoption of participatory design research methods for web and mobile based psychiatric interventions. Based on the review of the literature, the inclusion of participatory design research helps to make research more relevant and acceptable. From our review, it is apparent that such methodologies have not been utilised to assist in the conceptualisation of web and mobile-based interventions for individuals with addictive disorders. There is a potential utility to consider such methods, particularly for attention or cognitive bias modification for several reasons. The paradigms (that of the visual probe task for example) typically requires the individual to undertake onerous repeats of the trials to assess and to modify underlying attentional biases. This results in decreased intrinsic motivation to train. Participatory research methods could be used to better explore individual participants’ perception about the tasks and how best to increase their intrinsic motivation to train. Moreover, whilst methods such as that of gamification have been applied to attention bias modification as an attempt to address motivation to train, it is apparent from the previous review [31], that not all studies have reported its effectiveness and success. Participatory design methods could help examine participants’ perspectives towards gamification in attention bias modification interventions. This will help to better any future research that seeks to incorporate gamification strategies into conventional attention bias modification interventions. 

Most of the technological solutions to date have largely been created via a “top-down” method of health production, in which only organizations, pharmaceutical companies and academic centers are consulted [33]. This approach typically assumes that organizations and healthcare professionals are experts and understand the needs of the patients [33]. This approach and the importance of adopting participatory design methods was made apparent in 2014, when a patient created a mobile technological system for Type I Diabetes, which was eventually widely adopted in healthcare systems [33]. This demonstrates the ability of patients themselves in harnessing technologies to co-create their own healthcare interventions that meets their specific needs, but also how a network of patients could collaborate and conceptualize a technological solution. More importantly, the prior patient-led project demonstrates the importance of understanding patients’ perspectives and their needs during the conceptualization of a technological solution. Considering interventions in psychiatry, Dekker et al. [34] have similarly highlighted the importance of involving end-users in the design and decision-making process when conceptualizing and developing games, as an intervention for depression and anxiety. Their recent review revealed that 50% of identified games have had included inputs from participants [34]. Co-design approach was used in the development of 30% of these games [34]. 

In the area of attention bias modification interventions, Zhang et al. [28] have highlighted in their review there being more commercial applications (a total of 17 applications), whilst there have only been eight prior published studies. Thus, for attention and cognitive bias modification applications, a typical top-down approach in conceptualization is still being utilized, with limited involvement of the participants. In our prior literature review, it was highlighted that co-creation and design workshops are the commonest participatory research design methods being used in the conceptualization of both web and mobile based healthcare interventions. As we have already highlighted, participatory research methods could help in understanding the perspectives of participants; and help in the identification of appropriate gamification strategies that could be considered to better the conventional bias modification paradigm. Co-design workshops also enable participants to pro-actively co-create and design the new attention or cognitive bias modification application. 

## 7. Conclusions

It is clear from the research literature that participatory design methods are increasingly used in the conceptualization of both web and mobile based psychiatric interventions. It is of importance for researchers who are developing or evaluating web or mobile based attention or cognitive bias-based interventions to consider the inclusion of such research methodologies, as it could help to address the inherent limitation of bias modification training, that of the motivation to train and better the existing applications for more effective modification of attention and cognitive biases in individuals. 

## Figures and Tables

**Table 1:** Characteristics of the Included Studies.

Studies	Participants	Psychiatric Condition	Participatory Design Research (PDR) Method & Method of Analysis	Rationale for inclusion of Participatory Design Research Methods	Main Findings/Results	Key Limitations (Specific for Methods)
Gordon, M, et al. (2016) [15]	Core group of participants (n = 17), which is made up of: - Patients with history of depression in pregnancy (n = 4) - Prenatal providers (n = 2) - Social workers/care managers (n = 2) - Mental health specialists (n = 2) - Clinic administrator (n = 1) - Support staff (n = 3) - Research staff (n = 2) - Programmer (n = 1)	Perinatal Depression	Iterative participatory design strategy: - Meeting occurred monthly in a waiting area of the prenatal centre immediately following the regular workday and lasted 2 h. - Meetings were focused on generating ideas and providing feedback on brief pilot studies. Meetings facilitated by the research team and involved brainstorming, problem-solving, role-playing, and feedback on content. Method of Analysis: - Rapid cycle iterative approach. - At each of the meetings, progress was reviewed, feedback was provided, the pilot software was tested, and it helped to guide the specific recommendations for the next steps. - Live-action videography was used. - Animated guides were produced by means of a commercial software.	Aim to develop e-health tools geared to the particular support needs of low-income, ethnic/racial minority women at risk of perinatal mental disorders (PMD).	3 Applications were developed, the MyGamePlan suicide prevention mobile app for iPhone, a tablet-based screening tool for PMD and a patient decision aid (PtDA) for supporting treatment decisions for depression in pregnancy by women and their clinicians.	Multistakeholder participatory design groups take time to establish.
O’Connor S et al. (2016) [10]	Total number of participants (n = 16); in-depth focus group (n = 10) and interviews (n = 6). Participants included that of dementia patient-carer dyads, an occupational therapist, a project manager and a software engineer.	Dementia	Qualitative exploratory case study design: - In-depth focus (n = 10) and interview (n = 6) with people involved in the co-design was conducted. Method of analysis: - Data was analyzed thematically using the framework approach.	To explore the barriers experienced by all participants during the co-design of the My House of Memories application, to ensure that the future production of mobile technology is more effective.	The study highlighted the lack of digital literacy knowledge and skills among people with dementia and their carers. Inaccurate perceptions of how people with dementia or carers would use mobile technology. Individuals in the later stages of dementia struggled to take part in the workshops and compromises also had to be made to the design and functionality of the mobile app.	Not mentioned
Ospina-Pinillos, L, et al. (2018) [11]	18 young participants & 10 youth health professionals participated in stage 1. The group included 4 psychologists, 2 occupational therapists, 1 medical student, 1 general practitioner, 1 social worker and 1 Aboriginal youth worker. 9 young people and youth health professionals in Stage 2. The young health professionals included 3 psychologists and 1 occupational therapist. 6 people participated in Stage 3. This also included 1 clinical psychologist, 1 psychology student and 4 young people.	Not specified For the creation of a web-based mental health clinic	Four Participatory Design Workshops heldEach workshop was followed by a knowledge translation session. At the end of the cycle, the alpha prototype was built with one round of one-on-one end user consultation sessions conducted. PD Workshop (Phase 1): Transition of knowledge and idea generated during workshops to produce mock-ups of webpages Phase 2: Rapid prototyping with one-on-one consultations with end users Phase 2: Rapid prototyping and user acceptance testing Methods of Analysis: Not specified	To help develop a Mental Health eClinic to improve timely access to, and better quality, mental health care for young people	Helped in revealing the importance of five key components for the Mental Health eClinic: a welcoming home page with a visible triage system; a comprehensive physical and mental health assessment; a detailed dashboard of results; a booking and a video visit system; and the generation of a personalized well-being plan that includes links to evidence-based, and health professional-recommended, apps and etools.	Nothing specific to PDR
Owens, C. et al. (2010) [16]	Eight mental health service users and one carer. Three liaison psychiatry team members.	Self-Harm	6 participatory workshops First workshop: Full day, designed to introduce people to the project, enable the various stakeholders to understand and feel comfortable with one another. Time was spend exploring the meaning and functions of self-harm and attitudes towards prevention. Acceptability and merits of text messaging were discussed. Methods of Analysis: - Principal task in Workshop 1: Generate a catalogue of potential messages that could be market tested. - Workshop 2: Focus group, with additional participants with histories of self-harm. Purpose: validating and rating the messages generated in Workshop 1 - Workshop 3: Retest of a selection of messages, followed by a free-ranging discussion - Workshop 4,5,6 involved stimulating, evaluating and refining the intervention.	Involving potential recipients at the design stage may be particularly important when the intervention is intended to bring about behavioural, as opposed to biochemical, change. This study reports on the challenge of working with a group of people with relevant lived experience to develop a text-messaging intervention to reduce repetition of self-harm.	Service users rejected both the idea of a generic, one-size fits all approach and that of audience segmentation. Text messages could be safe and effective only if individualized.	Workshop participants were not involved in every aspect of decision making. Difficulties in keeping the same group of participants due to their fluctuations in the mental state. Researchers need to keep restating the aims of the project and re-establishing consensus at each workshop. The iterative nature of the participatory process meant that plans had to be revised in response to the findings from each session.
Peters, D. et al. (2018) [17]	Male workers, invited to participate via email announcements in 2 male-dominated organizations in Australia. Total of 60 workers, Mean age 47 years old, 97% of the participants reported the use of a mobile phone	Not specified General Mental Health Well-being of Males in the Workplace	Participatory Design Workshop Activity-based workshop, 2.5 h each, were carried out with groups of participants (minimum of 5 and maximum of 17 participants in each group). Activities included individual reflection, collaborative ideation, and paper prototyping. Data collated included participant-generated artefacts from each of the activities, collection of feature ideas on sticky notes, field notes and audio recording of each workshop. Methods of Analysis: Theoretical thematic analysis	To address the difficulties in engaging men with mental health support by presenting qualitative results of their perceptions, preferences and ideas related to mental health technologies and providing an example of how a research-based app for mental health could be designed.	Workers do have a set of features, languages and style preferences that they feel would be essential in any future mental health app. Perceived stigma against the term “mental health” with terms like “mental fitness” being preferred. Range of specific features including mood tracking, self-assessment and “mood boost” tools were highly valued in addition to characteristics such as brevity, minimal on-screen text and a solutions-orientated approach.	Self-selection bias – those who volunteered for the study are likely to represent those who were more willing to discuss mental health issues.
Whitehouse, S.R. et al. (2013) [18]	A design student from Emily Carr University of Art and Design (ECUAD) undertook the initial co-creative research and subsequent preliminary development of TickiT with youth from the Youth Advisory Committee (12–20 years old, n = 8) Subsequent co-creation sessions were held with adolescents in a high school (n = 16) and a 1st year design class at ECUAD (n = 24).	Not specified Mental health issues in Youth	Total of three 2-h co-creation sessions First group co-creation session – youths brainstormed about the concept of psychosocial screening, to determine strategies that could be followed to align their ideas with those of health professionals. Focus groups were set up to discuss youth perceptions of content and language from the questionnaire. Subsequent sessions youths were provided with paper PDF copies of version of questions reflecting revisions suggested from their previous feedback sessions. These were presented on colour templates with icons for responses. Youths were asked to interact with the interface, provide comments on the copies and discuss their feelings about the User interface. Methods of Analysis: - Not specified	To improve the uptake of psychosocial screening among youths	This study described the early developments of the TickiT eHealth platform that was initially designed to engage the patient, enhance the relationship between patient and provider and improve efficiency. Youths gave an overall positive response regarding the concept of the tool. Some were concerned about privacy. Suggestions regarding specific design constructs. Identified problems with the text, simplified questions. Concerns raised with technical issues.	Lack of support for long-term research collaborations between industry and academic institutions. Industry timelines are inconsistent with academic timelines.
Wiljer, D. et al. (2017) [19]	65 post-secondary students	Not specified. Creation of a mental health platform	Co-creation involved student development teams, hosting a hackathon, conducting focus group and evidence-based workshops and student advisory groups. Key components of the co-design workshop: Student driven development teams; Crowd-sourcing/data workshops; Hackathon; Knowledge transition and engagement strategies. Methods of Analysis: - Not specified	Co-design could strengthen youth buy-in, increased the efficacy, usability and sustainability of the intervention, generate innovative and creative design concepts and foster youth empowerment, skill and capacity building.	This project has demonstrated the benefits and challenges of co-creation in the development of a crowd-sourcing platform for students seeking support for their mental health.	Some challenges in maintaining individual engagement.
